# Supplementary material for: Diagnostic and Prognostic Power of Active DNA Demethylation Pathway Intermediates in Acute Myelogenous Leukemia and Myelodysplastic Syndromes
Source: Cells. 2022 Mar 4;11(5):888. doi: 10.3390/cells11050888 (PMC8909098; doi:10.3390/cells11050888)
Supplement: Supplementary file 1 [file cells-11-00888-s001.zip › cells-1560411-supplementary.pdf]

Supplementary Materials

1. Supplementary Methods

1.1. A detailed description of the determination of the epigenetic modifications in urine.

2D-UPLC-MS/MS 2D-UPLC-MS/MS was used for the epigenetic modification analysis of urine samples (with the exception of 5-hmUra). Urine samples were spiked with a mixture of internal standards at a 4:1 volumetric ratio. The 2D-UPLC–MS/MS system consists of a gradient pump and autosampler for one-dimensional chromatography, and a gradient pump and tandem quadrupole mass spectrometer with a UniSpray ion source was used for two-dimensional chromatography. Both systems were coupled with a column manager equipped with two programmable column heaters and two 2-position 6-port switching valves. The at-column dilution technique was used between the first and the second dimensions to improve the retention in the trap/transfer column. The sample molecules were then adsorbed to the packing material as very narrow bands that could be eluted with well-resolved, small-volume peaks. A diluting stream of water (0.5 mL/min) was pumped with a Waters 515 isocratic pump and mixed with the first-dimension column effluent using a UPLC low-dead-volume tee valve. The following columns were used: CORTECS UPLC T3 Column (1.6 µm, 3 mm × 150 mm) with a CORTECS T3 VanGuard precolumn (1.6 µm, 2.1 mm × 5 mm) for the first dimension, a Waters ACQUITY UPLC CSH C18 (1.7 µm, 2.1 mm × 100 mm) for the second dimension, and a Waters XSelect CSH C18 column (3.5 µm, 3 mm × 20 mm) as the trap/transfer column. The chromatographic system was operated in heart-cutting mode, which means that selected portions of effluent from the first dimension were loaded onto the trap/transfer column by 6-port valve switching, which served as an "injector" for the second dimension of the chromatography system. Mass spectrometric detection was conducted with a Waters Xevo TQ-S tandem quadrupole mass spectrometer equipped with a UniSpray ionization source. The following common detection parameters were used: source temperature, 150°C; nitrogen desolvation gas flow, 1000 L/h; nitrogen cone gas flow, 150 L/h; desolvation temperature, 500°C; and nebulizer gas pressure, 7 bar. Collision-induced dissociation was obtained with argon (6.0 at 3 × 10-6 bar pressure) as a collision gas. The instrument response to all compounds was optimized by the infusion of 10 µM genuine compounds dissolved in water (10 µL/minute) in the mobile phase A stream via the mass spectrometer fluidics system operating in the "mixed" mode using MassLynx 4.1 software IntelliStart feature. The chromatographic system was operated with MassLynx 4.1 software from Waters. Quantitative analyses were performed using the TargetLynx application. All samples were analyzed with three to six technical replicates. Due to the low sensitivity of the method used, the level of 5-hmUra was determined by high-performance liquid chromatography for pre-purification followed by gas chromatography with isotope dilution mass spectrometric detection (LC/GC–MS), as previously described [1,2].

1.2. A detailed description of the isolation of DNA and the determination of the epigenetic modifications in DNA isolates

Leukocytes were isolated from heparinized blood samples with Histopaque 1119 (Sigma) solution, according to the manufacturer's instructions, and stored at −80°C until analysis. The analyses were performed using a method described earlier by Gackowski et al. and Starczak et al. with some modifications [3,4]. Briefly, a pellet of frozen cells was dispersed in ice-cold buffer B (Tris-HCl (10 mmol/L), Na_2EDTA (5 mmol/L) and deferoxamine mesylate (0.15 mmol/L), pH 8.0). SDS solution was added (to a final concentration of 0.5%), and the mixture was gently mixed using a polypropylene Pasteur pipette. The samples were incubated at 37 °C for 30 minutes. Proteinase K was added to a final concentration of 4 mg/mL

and incubated at 37 °C for 1.5 h. The mixture was cooled to 4 °C, transferred to a centrifuge tube with phenol:chloroform:isoamyl alcohol (25:24:1), and vortexed vigorously. After extraction, the aqueous phase was treated with a chloroform:isoamyl alcohol mixture (24:1). The supernatant was treated with three volumes of cold 96% (v/v) ethanol to precipitate high molecular weight nucleic acids. The precipitate was removed with a plastic spatula, washed with ethanol and dissolved in Milli-Q grade deionized water. The samples were mixed with 200 mM ammonium acetate containing 0.2 mM $ZnCl_2$, pH 4.6 (1:1 v/v). Nuclease P1 (100 U, New England Biolabs) and tetrahydrouridine (10 μg/sample) was added to the mixture and incubated at 37°C for 3 h. Subsequently, 10% (v/v) NH_4OH and 6 U of shrimp alkaline phosphatase (rSAP, New England Biolabs) were added to each sample and incubated for 1.5 h at 37 °C. Finally, all the hydrolysates were ultrafiltered prior to injection. The DNA hydrolysates were spiked with a mixture of internal standards at a volumetric ratio of 4:1 to a final concentration of 50 fmol/μL: [D_3]-5-(hydroxymethyl-2-deoxycytidine (5-hmdC), [$^{13}C_{10}$, $^{15}N_2$]-5-formyl-2′-deoxycytidine, [$^{13}C_{10}$, $^{15}N_2$]-5-carboxy-2′-deoxycytidnie, and [$^{13}C_{10}$, $^{15}N_2$]-5-(hydroxymethyl)-2′-deoxyuridine. Chromatographic separation was performed with a Waters ACQUITY 2D-UPLC system with a photodiode array detector for the first dimension of the 2D-chromatography (used for quantification of the unmodified deoxynucleosides and 5-methyl-2′-deoxycytidine) and a Xevo TQ-S tandem quadrupole mass spectrometer (used for the second dimension of the 2D-chromatography to analyze 5-hmdC from the first dimension in positive mode, to assure better ionization at higher acetic acid concentrations). The at-column dilution technique was used between the first and second dimensions to improve the retention on the trap/transfer column. The following columns were used: a Waters CORTECS T3 column (150 mm×3 mm, 1.6 μm) with a precolumn for the first dimension, a Waters XSelect C18 CSH (100 mm×2.1 mm, 1.7 μm) for the second dimension and a Waters XSelect C18 CSH (20 mm×3 mm, 3.5 μm) column as a trap/transfer column. The chromatographic system was operated in heart-cutting mode, indicating that selected fractions of the effluent from the first dimension were loaded onto the trap/transfer column by 6-port valve switching, which served as the "injector" for the second dimension of the 2D-chromatography process. The flow rate for the first dimension was 0.5 mL/minute, and the injection volume was 2 μL. Separation was performed with a gradient elution for 10 minutes using a mobile phase of 0.05% acetate (A) and acetonitrile (B) (0.7-5% B for 5 minutes, column washing with 30% acetonitrile and re-equilibration with 99% A for 3.6 minutes). The flow rate for the second dimension was 0.3 mL/minute. The separation was performed with a gradient elution for 10 minutes using a mobile phase of 0.01% acetate (A) and methanol (B) (1-50% B for 4 minutes, isocratic flow of 50% B for 1.5 minutes, and re-equilibration with 99% A until the next injection). All samples were analyzed with three to five technical replicates, of which the technical mean was used for further calculation. Mass spectrometric detection was performed using a Waters Xevo TQ-S or TQ-XS tandem quadrupole mass spectrometer equipped with an electrospray ionization source. Collision-induced dissociation was obtained using argon 6.0 at 3 × 10-6 bar pressure as the collision gas. Transition patterns for all the analyzed compounds and the specific detector settings were determined using the MassLynx 4.1 IntelliStart feature set in a quantitative mode to ensure the best signal-to-noise ratio and a resolution of 1 at MS1 and 0.75 at MS2.

1. Rozalski, R.; Gackowski, D.; Skalska-Bugala, A.; Starczak, M.; Siomek-Gorecka, A.; Zarakowska, E.; Modrzejewska, M.; Dziaman, T.; Szpila, A.; Linowiecka, K.; et al. The urinary excretion of epigenetically modified DNA as a marker of pediatric ALL status and chemotherapy response. *Sci Rep* **2021**, *11*, 21345, doi:10.1038/s41598-021-00880-9.
2. Rozalski, R.; Gackowski, D.; Siomek-Gorecka, A.; Starczak, M.; Modrzejewska, M.; Banaszkiewicz, Z.; Olinski, R. Urinary 5-hydroxymethyluracil and 8-oxo-7,8-dihydroguanine as potential

biomarkers in patients with colorectal cancer. *Biomarkers* **2015**, *20*, 287-291, doi:10.3109/1354750x.2015.1068860.
3. Gackowski, D.; Starczak, M.; Zarakowska, E.; Modrzejewska, M.; Szpila, A.; Banaszkiewicz, Z.; Olinski, R. Accurate, Direct, and High-Throughput Analyses of a Broad Spectrum of Endogenously Generated DNA Base Modifications with Isotope-Dilution Two-Dimensional Ultraperformance Liquid Chromatography with Tandem Mass Spectrometry: Possible Clinical Implication. *Anal Chem* **2016**, *88*, 12128-12136, doi:10.1021/acs.analchem.6b02900.
4. Starczak, M.; Gawronski, M.; Olinski, R.; Gackowski, D. Quantification of DNA Modifications Using Two-Dimensional Ultraperformance Liquid Chromatography Tandem Mass Spectrometry (2D-UPLC-MS/MS). *Methods Mol Biol* **2021**, *2198*, 91-108, doi:10.1007/978-1-0716-0876-0_8.
